# Chitosan Nanoparticles-Insight into Properties, Functionalization and Applications in Drug Delivery and Theranostics

**DOI:** 10.3390/molecules26020272

**Published:** 2021-01-07

**Authors:** Jhanvi Jhaveri, Zarna Raichura, Tabassum Khan, Munira Momin, Abdelwahab Omri

**Affiliations:** 1 SVKM’s Dr. Bhanuben Nanavati College of Pharmacy, Mumbai 400056, Maharashtra, India; jhaverijhanvi@gmail.com (J.J.); zarnaraichura@gmail.com (Z.R.); 2 Department of Pharmaceutical Chemistry, SVKM’s Dr. Bhanuben Nanavati College of Pharmacy, Mumbai 400056, Maharashtra, India; Tabassum.Khan@bncp.ac.in; 3 Department of Pharmaceutics, SVKM’s Dr. Bhanuben Nanavati College of Pharmacy, Mumbai 400056, Maharashtra, India; Munira.Momin@bncp.ac.in; 4 The Novel Drug & Vaccine Delivery Systems Facility, Department of Chemistry and Biochemistry, Laurentian University, Sudbury, ON P3E 2C6, Canada

**Keywords:** chitosan, nanoparticles, functionalization, drug delivery, theranostics

## Abstract

Nanotechnology-based development of drug delivery systems is an attractive area of research in formulation driven R&D laboratories that makes administration of new and complex drugs feasible. It plays a significant role in the design of novel dosage forms by attributing target specific drug delivery, controlled drug release, improved, patient friendly drug regimen and lower side effects. Polysaccharides, especially chitosan, occupy an important place and are widely used in nano drug delivery systems owing to their biocompatibility and biodegradability. This review focuses on chitosan nanoparticles and envisages to provide an insight into the chemistry, properties, drug release mechanisms, preparation techniques and the vast evolving landscape of diverse applications across disease categories leading to development of better therapeutics and superior clinical outcomes. It summarizes recent advancement in the development and utility of functionalized chitosan in anticancer therapeutics, cancer immunotherapy, theranostics and multistage delivery systems.

## 1. Introduction

**Figure 1:**
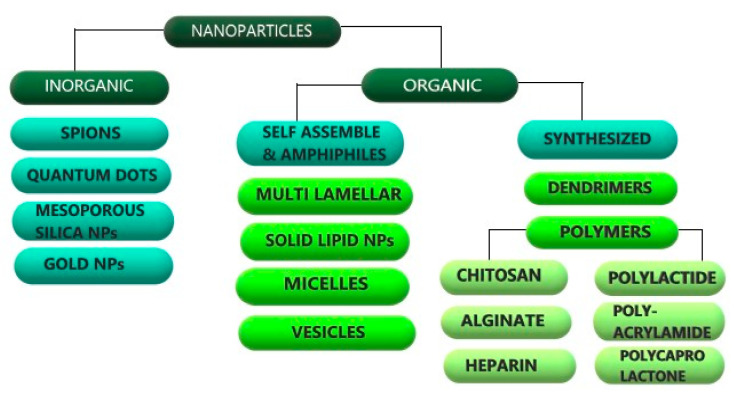
Classification of NPs.

Conventional drug delivery systems possess major limitations of non-specific delivery, poor dissolution, reduced absorption leading to poor bioavailability and sub-optimal plasma drug concentrations. These may result in side effects due to accumulation of drugs in other organs, a consequence of non-specific delivery. Novel drug delivery systems have been developed and intensively researched as a means to overcome the limitations of conventional drug delivery systems for a diverse category of drugs leading to improved patient compliance and better therapeutic and clinical outcomes. Modulating delivery of existing drugs to maximize their efficacy is a relatively simple and feasible alternative to new drug discovery as discovery of new drugs is an expensive, complex and time intensive process. Nanotechnology provides the advantage of improved efficacy and lower side effect profile. It involves redesigning and functionalizing materials at a molecular level to confer distinct attributes of larger surface area offering higher drug payloads, antibacterial activity and antifungal activity, high surface hardness (in nanocomposites), electrical properties (in nano-filled coatings) and magnetic properties that can be harnessed for versatile applications in healthcare. The increased interest and attraction of these nanomaterials is because of their unique optical, dielectric and surface plasmon resonance properties that makes them amenable to diverse applications in electronics, mechatronics, medicine and pharmaceuticals. The expanding landscape of nano-based delivery systems includes nanoparticles, nanospheres, nanotubes, nanosensors, nanocrystals, nanorobots, nanocapsules. Nanoparticles (NPs) act as nanocarriers carrying high drug payloads to the target site along with controlled release. NPs being small in size (1–100 nm) can easily pass through the finest blood capillaries and avoid phagocytic clearance thereby prolonging its plasma half-life and providing sustained drug release. NPs are used to deliver biomolecules such as proteins, peptides, enzymes, genes, vaccines in addition to small molecule drugs via versatile delivery routes like intranasal, oral, vaginal, buccal and pulmonary. They are extensively used in anticancer, gene therapy, immunotherapy, tissue regeneration, metabolic disorders, neurological disorders and theranostics. NPs are broadly classified into inorganic and organic NPs (Figure 1). Chitosan is a natural polymer that is widely researched due to its immense potential in fabricating nanocarrier systems. This review presents an insight on the properties, functionalization and applications of Chitosan NPs in drug delivery along with their pharmacokinetic and safety profiles.

### Chitosan

Chitosan is a Nature-derived mucopolysaccharide, closely related to cellulose with an acetyl amino group in place of a hydroxyl group at the C-2 position. It is obtained by deacetylation of chitin, a major component of the exoskeletons of insects, crustaceans (like shrimps) and fungal cell walls. Chitin is a polymer of β-(1,4)-*N*-acetyl-D-glucosamine which on deacetylation in an alkaline environment yields chitosan, a polymer of *N*-acetylglucosamine and D-glucosamine units [1].

**Figure 2:**
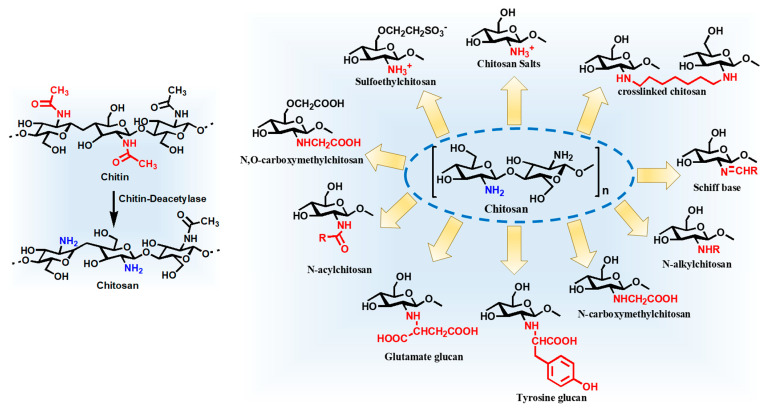
Synthesis of chitosan from chitin and structures of some of functionalized chitosan derivatives.

Chitosan has suitably positioned functional groups that confer specific properties to this polysaccharide. The presence of amino group at C-2 position of the glucosamine unit strengthens the functional and structural properties of chitosan. This amino group represents its cationic nature and imparts inherent properties of wound healing, antimicrobial activity and most importantly mucoadhesiveness making it a good carrier material in drug delivery systems. It has a pKa of 6.5, it is insoluble in water but soluble in acidic solutions. It is protonated and polycationic in nature and forms complexes with diverse anions like lipids, proteins, DNA, alginate, pectin and poly(acrylic acid). The physicomechanical properties (solubility, toxicity, hydrophobicity) of chitosan depend on the degree of deacetylation and molecular weight of chitosan depending on the source of chitin [2,3]. The attractiveness of chitosan lies in it being biodegradable, biocompatible, simple, stable, nontoxic and an intensively researched polymer looking at the magnitude of papers available on chitosan functionalization and applications across diverse sectors. It can be modified by chemical or enzymatic functionalization strategies. Functionalization of the amino and hydroxyl groups results in a range of *N*-modified, *O*-modified and *N*,*O*-modified chitosan resulting in diverse attributes and enhanced biological activity. Quarternized, *N*-alkyl-/*N*-benzylchitosan and phosphorylated chitosan increases its antimicrobial activity and solubility, thiolated chitosan improves its mucoadhesive properties. The nonselective *N*,*O*-modified chitosan derivatives are synthesized using electrophilic reactants like alkyl halides, acids and isocyanides. Selective *O*-modified chitosan derivatives are obtained by using acids like H_2_SO_4_ or MeSO_3_H, the acid protonates the amine group leaving the hydroxyl group free to undergo the reaction and *N*-modified derivatives are obtained by protecting the hydroxyl functional group [4]. Figure 2 depicts the synthesis of chitosan from chitin and the structures of some widely used functionalized chitosan derivatives [2,5,6].

## 2. Properties of Chitosan

**Figure 3:**
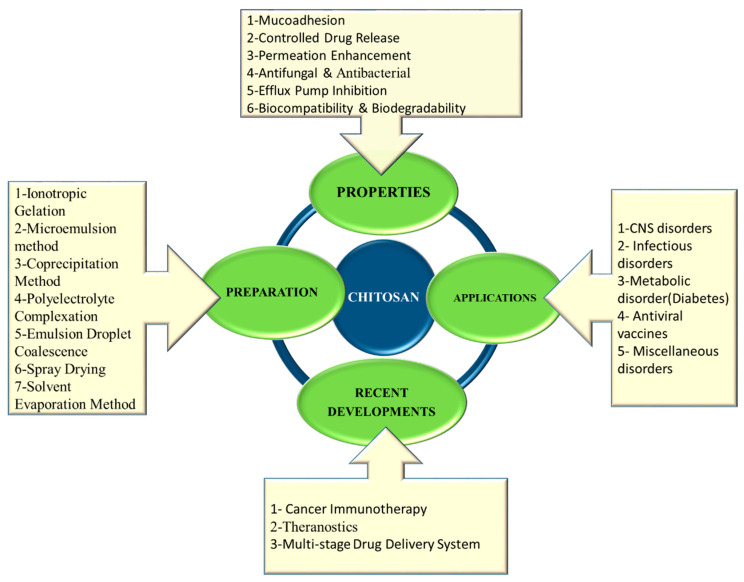
Multifaceted chitosan in drug delivery.

The attractive properties of chitosan promote its utility across diverse sectors of pharmaceuticals, agriculture, food industry, and bioengineering industry and are discussed here. Figure 3 depicts the properties of chitosan, methods of preparation of chitosan NPs and applications across various drug delivery platforms.

### 2.1. Mucoadhesion

The mucoadhesive property of chitosan is attributed to its cationic character. The mucous membrane is composed of mucin glycoprotein and has anionic functionalities in the form of sialic acid and sulfonic acid. The cationic group in chitosan and these anionic acids in the mucous results in ionic interactions conferring mucoadhesive attributes to chitosan. The mucoadhesion increases with the degree of deacetylation and its molecular weight and decreases with an increase in crosslinking. This attribute promotes its adherence to the surface of gastro-intestinal tract and upper respiratory and is instrumental in retaining this carrier for prolonged time achieving sustained release of drug payloads. Thiolation leads to increase in mucoadhesion of chitosan by creating strong interaction (disulphide bonds) between the thiol groups and the cysteine region of the mucous glycoprotein. The functionalized derivatives of chitosan: trimethyl chitosan, carboxymethyl chitosan have been reported to enhance the mucoadhesive properties [7].

### 2.2. Controlled Drug Release

The ability of chitosan to form ionic crosslinks leads to formation of stable complexes releasing the drug over a prolonged period of time conferring controlled drug release. This is beneficial for drugs that show suboptimal plasma levels and oral administration and have to be given parentally. It is also useful for carrying drugs that are susceptible to metabolic degradation in the gastro-intestinal tract assuring enhanced efficacy and patient compliance [8].

### 2.3. Permeation Enhancement

Chitosan being positively charged, interacts with the mucus membrane and opens the tight junctions between the cells (by reducing the transepithelial electrical resistance) promoting passage through the mucosal cells and enhancing drug permeation. This is beneficial for hydrophilic and high molecular weight compounds like proteins and peptides. Modified chitosan like thiolated and trimethyl chitosan show improved permeation enhancement effect than chitosan. Trimethylchitosan exhibits high water solubility and is biodegradable, biocompatible and more bioadhesive then chitosan, which makes it an attractive NP carrier. It occurs due to redistribution of F-actin of cytoskeleton, as observed for permeation of insulin in the intestinal mucosal cell line Caco-2. Trimethylchitosan-loaded resveratrol showed high permeation and higher cellular uptake due to high surface positive charge on the trimethyl chitosan NPs [9,10].

### 2.4. Antibacterial and Antifungal Activity

Chitosan exhibits antibacterial activity due to the positively charged amine groups that interact with the negatively charged components of bacterial cell wall. It is active against both gram-positive and gram-negative bacteria and is used in biomedical and clinical science to avoid bacterial colonization. It possesses antifungal activity wherein the mechanism of action involves morphogenesis of the cell wall, directly interfering with fungal growth. Low molecular weight chitosan can pass through cell membrane and interact with DNA to interrupt their functions. The antibacterial activity of chitosan depends on the pH of the environment (low pH is preferred since amine groups get protonated and enhance interaction with negatively charged bacterial cell surfaces), type of pathogen, degree of deacetylation, molecular weight and concentration. The gap of discovering new alternatives with regards to the emerging existing resistance to clinically prescribed antibiotics can be overcome by smart scientific exploitation and application of these chitosan properties. The antibacterial activity of chitosan NPs was examined against all strains of *N. gonorrhea*, including those resistant to multiple antibiotics, the results indicated chitosan NPs to inhibit growth with minimum inhibitory concentration of 0.16 to 0.31 mg/mL and a minimum bactericidal concentration of 0.31 to 0.61 mg/mL and no significant cytotoxicity [11,12].

### 2.5. Biocompatibility and Biodegradability

Biocompatibility of a material is an indication of the material being nontoxic and non-immunogenic to biological tissues. Chitosan exhibits very good biocompatibility because of its structural and functional resemblance to glycosaminoglycans present in the extracellular matrix of the human body and quickly forms hydrogels through crosslinking methods. It is easily degraded by in vivo lysozyme, chitinases and colon residing bacteria by virtue of the cleavage of glycosidic linkage in its structure. These properties can prove extremely beneficial for development of biocompatible and biodegradable drug delivery systems [13].

## 3. Preparation of Chitosan NPs as Nanocarriers

Chitosan NPs are synthesized using the ‘bottom up’ method, ‘top down’ method or some combination of both techniques. The “bottom up’ methods include ionotropic gelation, microemulsion method, solvent evaporation, polyelectrolyte method and the ‘top down’ methods include milling, high pressure homogenization and ultrasonication [4].

### 3.1. Ionotropic Gelation Method

This method involves crosslinking the cationic chitosan amino groups to a polyanionic crosslinker. Aqueous acidic solution of chitosan is prepared and added dropwise in tripolyphosphate (TPP) solution with continuous stirring at a constant rate. TPP being anionic, crosslinks with chitosan yielding chitosan-TPP NPs. This complex is used to entrap and carry drugs and these nanocarriers can be subsequently developed into suitable delivery systems. Chitosan-NaF NPs are reported to be prepared using this technique [14]. This method is simple, mild and easy, the use of aqueous medium eliminates the hazards and toxicities associated with the use of organic solvent. The NPs prepared by this method have the limitation of poor mechanical strength [15].

### 3.2. Reverse Micellar Method/Microemulsion Method

This method involves use of four components- polymer, surfactant, crosslinker (most commonly used is glutaraldehyde) and an organic solvent (*n*-hexane, toluene). It involves preparation of surfactant solution in a suitable organic solvent, preparation of polymer and crosslinker blend which is added to the surfactant mixture. The principle of crosslinking is based on the Schiff reaction and involves mixing the two solutions in the solvents, followed by removal of excess surfactant using calcium chloride yielding the desired polymer-crosslinker NPs [16].

### 3.3. Co-Precipitation Method

In this method, chitosan solution is prepared in a low pH acidic solution, a high pH solution like ammonium hydroxide is added resulting in highly monodisperse NPs. Chitosan-coated iron oxide NPs are reported to be prepared using this method [17].

### 3.4. Emulsion-Droplet Coalescence Method

This method involves both crosslinking and precipitation. Here two emulsions are prepared: (a) aqueous solution of chitosan along with the drug is added in liquid paraffin oil and stirred at a constant speed to give water/oil emulsion. (b) An aqueous solution of chitosan in sodium hydroxide is mixed in paraffin oil giving a second water/oil emulsion. The two emulsions are subsequently mixed with high speed stirring resulting in collision of droplets of the emulsions giving rise to coacervates, followed by centrifugation and filtration to yield chitosan-drug NPs [18]. Dexibuprofen NPs are reported to be prepared by this method for treatment of rheumatoid arthritis [19].

### 3.5. Polyelectrolyte Complexation Method

This method involves electronic interaction between positively charged amine groups of chitosan and negatively charged anions like carboxyl group of alginate or dextran group of dextran sulphate leading to charge neutralization. Chitosan solution is prepared in acetic acid and mixed with the anionic solution at room temperature under continuous stirring resulting in charge neutralization and self-assembly of the polyelectrolyte complexes [20,21]. Chitosan-guar gum NPs are reported to be prepared by this method for use in bone regeneration therapy [22]. Insulin loaded NPs are reported to be prepared by alginate ionotropic pre-gelation followed by polyelectrolyte complexation with chitosan for use in diabetes [23].

### 3.6. Solvent Evaporation Method

In this method, two different polymer solutions are prepared in volatile solvents, followed by addition of the drug to this mixture, and subsequent evaporation of the solvent to yield precipitated NPs. Repaglinide-loaded chitosan NPs are reported to be prepared using this method for use in diabetes [20].

### 3.7. Spray Drying Method

This is a well-known technique that produces powders, agglomerates and granules from a mixture of drug and excipient solutions or suspensions. In this method, chitosan is dissolved in acetic acid solution, to this drug solution and a suitable crosslinking agent is added, the resulting solution/dispersion is then atomized using a hot stream of air and this atomization leads to the formation of small droplets which on evaporation of solvent yield NPs. Spray dried inhalable powder containing nanoaggregates for pulmonary delivery of anti-tubercular drugs are reported to be prepared by this method [24]. Table 1 presents a summary of recent research on development of chitosan NPs using these techniques serving as nanocarriers for drugs along with entrapment efficiency, release profile, advantages and limitations.

Ionotropic gelation (the most preferred method for preparation of NPs based on the recent literature) is based on electrostatic interaction between amine group of chitosan with a negatively charged group of anionic polymers. This charge-based interaction of negatively charged drug payloads are more amenable to ionic gelation method resulting in high drug encapsulation efficiency, low polydispersity index and optimum drug release leading to entrapment efficiency ranging from 32% to 97% and drug release profile ranging from 44% to 80% [58]. The second most common method emerges to be polyelectrolyte complexation, which involves crosslinking of chitosan with drugs, and results in slow drug release. The other methods used to a lesser extent based on literature evidence include solvent evaporation, coprecipitation and emulsion droplet method due to poor entrapment efficiency and low cargo release profile.

## 4. Drug Release from Chitosan NPs in Biological Fluids 

**Figure 4:**
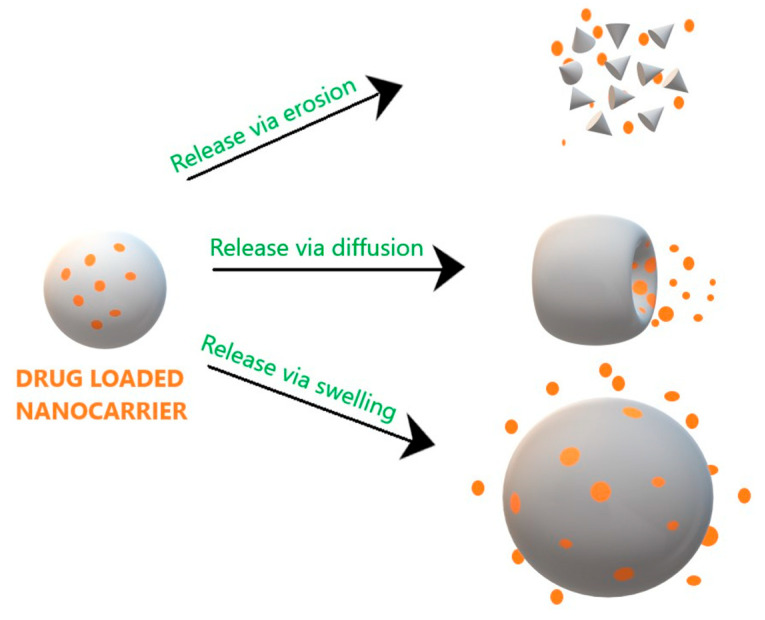
Drug release mechanism from chitosan NPs.

Drug release from NPs in biological fluids is one of the most important attributes impacting drug absorption, drug plasma levels and subsequent efficacy and is a measure of development of successful nanocarrier system [59]. The drug release from chitosan NPs occurs by three mechanisms as depicted in Figure 4.

### 4.1. Polymer Erosion/Degradation

It takes place in two ways, homogenous erosion wherein the complete polymer degrades and heterogeneous erosion wherein erosion of the polymer occurs from its surface to the inner core. Polymer degradation occurs in the surrounding media or presence of enzymes (by chemical reaction). Biodegradable polymers open due to cleavage of covalent bonds between them and bio erodible polymers degrade due to dissolution of linking chains without any change in chemical structure of the molecule [3,60].

### 4.2. Diffusion

The drug permeates from the core to the surrounding medium through a polymeric matrix. The drug diffuses from the matrix and through the cellular membrane and depends on the difference in concentration across the membrane, this mechanism of drug release is based on Fick’s law of diffusion.

### 4.3. Polymer Swelling 

Swelling of NPs is due to hydration and involves imbibing water into the polymer until the polymer dissolves and the polymer chains detangle. This disentanglement is usually characterized by a snake-like motion of the chain. This detangling creates a force that allows the release of drugs from the polymer matrix. The parameters affecting this mechanism include polymer solubility, polymer swelling rate, density of polymer chains, interaction of the polymer with the drug, particle size and drug-polymer-additive ratio. One of the important criteria is drug loading in the polymer nanocarrier, more the drug loading greater bursting effect and faster release of the nanocarrier and vice versa. Chitosan NPs exhibit change in properties in response to biological conditions like pH of the gastro-intestinal tract and cancer tissue helps in effective release of encapsulated drugs because of flexibility of chitosan NPs to protonate/deprotonate the polymer complex at altered pH conditions in biological fluids and tissue microspaces. Table 2 depicts the comparison of the three-drug release mechanism from chitosan NPs.

## 5. Properties of Chitosan NPs

The utility spectrum of chitosan NPs is predominantly determined by their morphological characteristics that include particle size, polydispersity index (PDI), zeta potential, surface charge and shape. These properties also modulate their biological activities. Their loading capacity and entrapment efficiency help to modulate drug payloads and subsequently drug related toxicity. The particle size of the NPs impacts drug related toxicity. The method of preparation plays a significant role in controlling these properties and even tailor them to develop chitosan NPs for specific applications in diverse sectors. The selection of the method is governed by the end-product requirements of particle shape, size, PDI, stability, release kinetics and toxicity. The ionotropic gelation method involves the use of negatively charged crosslinking agent sodium tripolyphosphate involved in an electrostatic interaction with chitosan to yield amphoteric ion-pair. This feature augments the protein adhesion attributes of the synthesized NPs. The chitosan-crosslinker molar ratio controls the mean diameter of the NPs and subsequently the release kinetics of the drug cargoes. The use of a weak acid like acetic acid as a solvent to dissolve the chitosan and the temperature of crosslinking impacts the size distribution while the stirring speed helps modulate the yield of NPs. Ionotropic gelation is a simple method utilizing mild reaction conditions that have minimal effect on the payload with researchers modifying this method to tweak the particle size of the NPs. A limitation of this method is low stability of the NPs in acidic medium and poor amenability for high molecular weight drugs [61,62,63]. The physicochemical characteristics of chitosan like crystallinity, solubility and degradation are governed by its molecular weight and degree of deacetylation that depends on selection of method and process parameters used during its production from chitin. 100% deacetylated chitosan exhibits maximum crystallinity and intermediate degree of deacetylation gives semi-crystalline chitosan. A higher degree of deacetylation corresponds to higher positive charge density resulting in increased solubility, increase in positive charge also increases interaction with the negatively charged mucin in mucus membrane augmenting its mucoadhesive property. Mima et al. reported a direct correlation of degree of deacetylation to the tensile strength of chitosan in wet state with no effect in dry state. Films prepared from high molecular weight chitosan showed higher tensile strength and tensile elongation as compared to low molecular weight chitosan. The degree of deacetylation inversely impacts the rate of degradation and is an important attribute modulated in the development of chitosan based scaffolds for tissue engineering applications to suit specific needs [64].

Rebbouh et al., prepared chitosan NPs by ionotropic gelation method for delivery of Aah II toxin isolated from *Androctonus australis* hector scorpion venom to fabricate a nanocarrier system for vaccine delivery. The resulting NPs were spherical with a smooth surface, the entrapment efficiency (EE) and loading capacity (LC) was reported to be 96.66% and 33.5% respectively. The Aah II-CNPs had a diameter of 208 nm, a PDI of 0.23 and a zeta potential of +30 mV. The low particle size permitted retention of NPs at the injection site with a depot effect. This depot could extend antigen presentation via sustained release and activation of antigen presenting cells. Encapsulation of Aah II reduced its toxicity and protected mice up to 10 LD50 and this nanocarrier system conferred peptide protection and prevention of its immediate and total release after administration. The developed system offered advantages of protection of antigens from harsh environment and enzymatic degradation; sustained release and targeted delivery along with desirable adjuvant functions. The in vivo studies demonstrated chitosan NPs to entrap sufficient amount of antigen without severe side effects to animals. In addition, Aah II-CNP induced lymphoid expansion showcasing the immunopotentiating properties of the NPs that improves their immunogenicity. The slow release of antigens achieved by the NPs is important to maintain plasma antigen concentrations below the threshold of toxicity, reducing the frequency of vaccine administration leading to minimal side effects ad these proved to be effective nanocarrier platform for the development of anti-envenoming therapy [47].

## 6. Pharmacokinetics of Chitosan NPs

Polymeric NPs offer improved efficacy of entrapped drugs like anticancer and peptide-based drugs that have very narrow therapeutic window or low bioavailability due to their inherent properties like molecular weight, size and shape and other morphological properties. The therapeutic efficacy and toxicity are mainly due to pharmacokinetic (PK) parameters, tissue deposition, metabolism and distribution in various organs. In addition to the physicochemical properties of the entrapped drug, the physicochemical properties of polymeric nanocarriers like composition (natural or synthetic polymers), electric charge on their surface along with their particle size, size distribution and surface chemistry play an important role in predicting the pharmacokinetic and bio distribution profile [65].

Chitosan NPs exhibit pharmacokinetic behavior similar to other polymeric NPs because the same principles of drug release apply as discussed in the earlier sections. Shabouri et al. conducted PK studies of chitosan NPs in beagle dogs to assess the bioavailability of cyclosporin-A (Cy-A) which was encapsulated in various NPs fabricated from polymers like chitosan, gelatin and sodium glycocholate (SGC). It was observed that the Cmax was markedly increased in the chitosan and gelatin NPs formulations while a reduced Cmax was observed with SGC NPs. A greater than 2-fold increase in AUC was observed with chitosan NPs as compared to SGC and gelatin. The surface charge on the selected polymers was responsible to the reported differences in PK parameters, i.e., chitosan being the cationic and the SGC being negative charged polymer. The negative charge of the SGC NPs hinders adherence to the intestinal mucus. It can be concluded from this study that the positive charge on chitosan NPs supports the increase in relative bioavailability. Xue et al. developed chitosan-based NPs of Bay 41–4109 for prolonging circulation time of the drug in blood. The study was conducted in rats and showed a 3.3-fold increase in Cmax, increased AUC and 4-fold increase in absolute bioavailability of chitosan NPs compared to the Bay 41–4109 suspension. It was observed that the NPs exhibited enhanced intestinal absorption of the drug due to improved interaction between the positive charge of chitosan with the negative charge of cell membranes. This characteristic led to prolonged drug release in the small intestine. Enoxaparin with a very low oral bioavailability was incorporated into chitosan NPs (Enx-Alg-CS-NPs), resulting in a 3-fold increase in AUC for oral enoxaparin (50 mg/kg in rats) representing 20% of the AUC achieved with intravenous dosing (1 mg/kg). This enhanced intestinal permeation of the drug in rats was attributed to the mucoadhesive property of the polymer. A number of similar studies have indicated the pharmacokinetic advantage of chitosan NPs over other polymeric NPs [66,67,68].

## 7. Safety and Toxicity of Chitosan NPs

Inorganic natural polymer-based NPs are generally considered safe; however, very limited studies are reported on the safety and toxicity of such NPs. Not only the type of polymers utilized play an important role in safety and toxicity of the nanocarrier but the size, shape and surface morphology also play a significant role in the safety and toxicity after oral or intravenous administration. Chitosan-based NPs are widely explored as carriers for gene/drug delivery, but the toxicity of chitosan NPs is not yet fully studied and understood to a large extent. However, reports available on various studies on chitosan NPs has revealed that chitosan exhibits low toxicity in various study models, (in-vitro and in vivo assays) via diverse routes of administration [60].

The application of chitosan as a nanoscale carrier, raises concerns and issues about associated nano toxicity. This nano toxicity of chitosan based nanomaterials could be associated with the size, shape, and electrokinetic potential (ζ-potential) of the nanoparticle [69].

Hu et al. studied chitosan NPs toxicity using a zebra fish model wherein chitosan NPs at different concentrations were incubated with zebrafish embryos with zinc oxide NPs as the positive control. Embryo exposure to chitosan NPs and ZnO NPs resulted in a concentration-dependent decrease in hatching rate and increase in mortality. Chitosan NPs at a size of 200 nm caused malformations, including bent spine, pericardial edema, and opaque yolk in zebrafish embryos. This experiment added new insights into potential associated toxicity of nature-derived polymeric NPs that are normally considered safe as they are produced from biodegradable materials. Such studies will help increase the understanding of the associated nano toxicity of drug nanocarrier delivery systems [70,71].

Another limitation of the available data on toxicity of NPs revolves around the duration of such studies. Most studies report the biocompatibility studies 2–4 days after intravenous administration in animal models. Reports are available for anticancer and anti-Alzheimer drug payloads which include various routes of administration, however, the toxicities determined by these type of studies, that use short periods of observation for long-circulating dispersed solutions may not be fully representative since neither the bio distribution nor the biodegradation processes are completed in such short periods [72,73,74].

## 8. Application of Chitosan NPs

The properties of chitosan NPs overcome the limitations of conventional drug delivery systems, the ease of preparation of chitosan NPs enables target specific drug delivery with improved drug efficacy. We present here an insight into the vast evolving landscape of diverse applications of chitosan NPs across disease categories leading to development of better therapeutics and superior clinical outcomes.

### 8.1. CNS Diseases

Chitosan NPs are widely used for encapsulation of hydrophilic drugs, nucleic acid, proteins and peptides because of their low permeability as well as for those drugs that undergo mucociliary clearance. The cationic nature of chitosan opens tight junctions of the blood brain barrier (BBB) via two pathways: (a) extracellular pathway and (b) intracellular pathways, facilitating drugs to cross the BBB and blood cerebrospinal fluid barrier. The mucoadhesive property of chitosan increases retention time and consequently increases its absorption and treatment efficacy. Usually, CNS disorder drugs are preferred to be delivered intranasally as this will allow better drug penetration in the brain and less systemic exposure.

The attributes of chitosan that enable efficient drug delivery to the brain include: particle size → influences endocytosis rate across the brain endothelial cells, high positive zeta potential → leads to good stability and absence of particle aggregation, high drug entrapment efficiency → due to good interaction between the drug and chitosan matrix, less prone to phagocytosis → results in reduced toxicity, modified chitosan surface like surfactant(tween 80, polysorbate 80) coated particles → efficient in target delivery to the brain. Table 3 presents application of chitosan NPs to carry drug payloads in CNS diseases.

Liu et al. reported that the intranasal drug delivery for epilepsy as an alternative to the conventional intravenous and oral administration of CBZ, developed by carboxymethyl chitosan NPs as a carrier for carbamazepine showed effective penetration through BBB and a sustained drug release with pronounced absorption.

Due to the unique properties of chitosan NPs, research on many other CNS disorders using chitosan NPs for the drug delivery was explored. In another study, to overcome the low bioavailability of gastrointestinal tract of sulpiride formulation an intranasal delivery of sulpiride-loaded chitosan NPs for schizophrenia was synthesized which showed improved solubility and stability assuring controlled drug release with minimum side effects. 

In a study for Parkinson’s disease, rotigotine-loaded chitosan NPs (RNPs) were fabricated and an in-vivo and ex-vivo study was performed to compare the effectiveness of RNPs vs. the rotigotine solution. It was observed that RNPs exhibited increased bioavailability and nasal residence. RNPs showed a sustained drug release profile thereby reducing the dosage regimen which was one of the major drawbacks of rotigotine solution (low plasma half-life).

Sunena et al. conducted a study to enhance therapeutic range of galantamine for Alzheimer’s disease, Galantamine-loaded thiolated chitosan NPs (GNPs) were synthesized and evaluated across nasal and orally administered galantamine solutions. GNPs showed significant results of recovery when evaluated on scopolamine induced amnesia in mice.

In another study for antipsychotic, olanzepine-loaded chitosan NPs were synthesized. The results of in vitro study showed optimum target specificity with a biphasic release pattern, an initial burst release followed by sustained release. Ex-vivo toxicity studies on human nasal epithelium cell line and goat nasal mucosa respectively exhibited no significant harmful effects of the nanoformulation. 

### 8.2. Infectious Diseases

The indiscriminate use of antimicrobial agents is associated with emerging drug resistance and the lack of new drug candidates in the drug discovery pipeline fuels extensive efforts to increase the efficacy of available drugs. The current drugs exhibit lower cellular uptake and varying plasma drug concentration leading to increased dosing frequency and associated side effects. In order to overcome these drawbacks, efforts are made to encapsulate the antimicrobial drugs in chitosan NPs. This encapsulation helps to reduce occurrence of drug resistance by increasing intracellular uptake, reducing efflux of drugs and preventing biofilm formation. The inherent antimicrobial property of chitosan, a consequence of its polycationic nature, allows interaction with negatively charged bacterial cell wall and cytoplasmic membranes, resulting in osmotic stability, membrane disruption and leakage of intracellular contents. It has the ability to enter the bacteria, inhibiting synthesis of m-RNA and proteins, a consequence of its binding affinity to bacterial DNA. Table 4 illustrates the application of chitosan NPs in delivery antimicrobial drugs.

Vancomycin-loaded *N*-trimethylchitosan NPs (VCM/TMC NPs) were fabricated by Xu et al., for chronic osteomyelitis and the results of in-vivo study indicated that osteoblasts exhibited an increase in alkaline phosphatase activity (biomarker for osteoblast differentiation) assuring target specific delivery and sustained drug release. Osteoblasts exposed to VCM/TMC NPs showed high cell proliferative ability. 

Kumar et al. prepared chitosan ascorbate NPs for vaginal delivery of amoxicillin. Their study on vaginitis, wherein the mucoadhesiveness of chitosan NPs was explicitly used to enhance amoxicillin loading into vaginal fluid and increase the mechanical resistance suitable for drug administration into the vaginal cavity indicated good benefits for the chitosan NPs.

Kumar et al. evaluated the efficiency of rifaximin-chitosan NPs for inflammatory bowel diseases. The results of this study demonstrated increased solubility, colon target specificity and increased therapeutic efficacy of rifaximin chitosan NPs.

Lee et al. prepared combination PLGA-lovastatin-chitosan-tetracycline NPs and evaluated them in beagle dogs having three walled defects. These NPs exhibited sequential release of tetracycline and lovastatin to effectively control local infection and promote bone regeneration in periodontitis. These therapeutic effects were enhanced by the biocompatibility, wound healing and antibacterial features of chitosan NPs demonstrating synergistic benefits. 

Sharma et al. prepared ketorolac tromethamine-loaded chitosan NPs in an effort to improve the ocular delivery of the drug used in the treatment of post-operative eye inflammation, allergic conjunctivitis and other ailments. The defensive mechanism of the eye, reduces the amount of bioavailable dose from conventional delivery systems. The in vitro drug release, stability studies, release kinetics and surface morphology showed increased bioavailability and ocular residence time, leading to better efficacy and therapeutic response.

### 8.3. Diabetes

Chitosan nanocarriers have been fabricated and for drugs used in diabetes and diabetes associated conditions with reasonable success. Table 5 indicates the encapsulation efficiency and zeta potential of some of the chitosan based nanocarrier systems.

Amphiphilic chitosan NPs modified by vitamin B_12_ were prepared to increase the oral bioavailability of scutellarin in Type 2 diabetes induced retinopathy. This study showed that these NPs exhibited higher permeation and cellular uptake in Caco-2 cells, thereby increasing its absorption. The in vivo study on Sprague–Dawley rats indicated improved bioavailability of NPs. These NPs reduced diabetes induced structural disorder of intra retinal neovessels in the retina and down regulated the expression of angiogenesis proteins (VEGF). Another study by Rong et al. reported increased neuroprotective effects of insulin-loaded chitosan NPs/PLGA-PEG-PLGA hydrogel on diabetic retinopathy in rats by subconjunctival injection [88].

A study by Veera et al. to overcome the low bioavailability and poor stability of curcumin, involved development of a novel nanohybrid scaffold-curcumin-chitosan NPs impregnated into collagen alginate scaffold for tissue regeneration application. In this nanohybrid, the NPs improved curcumin’s solubility and stability. The results indicated that the nanohybrid exhibited promising biocompatibility, anti-inflammatory, cell adhesion and proliferation, essential for tissue regeneration in diabetic wound healing. 

Rahbarian et al. studied insulin loaded thiolated *N*-triethylchitosan NPs via buccal delivery for diabetes. In-vitro study exhibited 98% insulin release from these NPs and ex-vivo study showed 96% permeability through buccal mucosa. The study concluded that this nanoparticulate system can be a good approach for buccal insulin delivery. Similarly, Omid et al. performed a study to fabricate novel glycyl-glycine and alanyl-alanine trimethyl chitosan conjugates for improved oral insulin delivery [89].

### 8.4. Antiviral Vaccines

Chitosan NPs-based vaccines are delivered via mucosal and systemic routes and are particularly useful in mucosal delivery because they promote absorption and mucosal immune response and in systemic administration chitosan acts as an adjuvant. Chitosan is widely used as a vaccine nanocarrier since it is protected from enzymatic degradation in the mucosal tissue and facilitates antigen uptake by mucosal lymphoid tissue thus, DNA mucosal vaccines are widely explored. It also demonstrates a potential for stimulating humoral and cellular immunity responses. Table 6 depicts chitosan NPs based antiviral vaccine delivery systems.

An effort was made to develop a vaccine for leishmaniasis using chitosan NPs containing whole leishmania lysate antigen (WLL) and soluble leishmania antigens (SLA). The aim of this study was to overcome the limitation of low efficacy due to the lack of a suitable adjuvant. The study however, did not show any significant difference in the activity of the NPs formulation with respect to induction of pure Th1-type immune response and did not fetch the desired outcome. Nevertheless, in another study conducted by Fernando et al. indicated that the developed IBV vaccine (containing BR-I genotype strain encapsulated in chitosan NPs (IBV-CS) was a suitable candidate to induce marked IFNγ gene expression and production of IgA and IgG anti-IBV antibodies across avian infectious bronchitis virus (IBV) [93].

Dhakal et al. evaluated mucosal immunity and protective efficacy for influenza vaccine on pigs. The study involved incorporation of killed SwIAV H1N2 (δ-lineage) antigens (KAg) in chitosan NPs. Pigs vaccinated by this nanovaccine increased IgG antibodies and elicited strong cross-reactive mucosal IgA and cellular immune responses by increasing frequency of cytotoxic T-lymphocyte, IFN-γ secretion and lymphocyte proliferation. This was observed against all Influenza A virus strains.

Jesus et al. evaluated poly-ϵ-caprolactone/chitosan NPs for hepatitis B. NPs showed adjuvant effect by secreting strong anti-hepatitis B surface antigen IgG1 isotype induced IFN-γ and IL-17 secretions. Induction of Th1/Th17 mediated cellular immune response was also observed.

### 8.5. Miscellaneous Diseases

Chitosan nanocarriers have been reported to be used for delivery of drugs in diseases not discussed earlier like hypertension. Table 7 depicts the utility of chitosan NPs in miscellaneous diseases.

Biopeptides were encapsulated in chitosan NPs by Saari et al. to improve the efficacy of orally administered anti-hypertensive ACE-inhibitory biopeptides. This nanoencapsulation circumvented the enzymatic degradation caused by gastrointestinal barriers. In vitro study on spontaneously hypertensive rats showed controlled release of the biopeptides. This study also demonstrated dose-dependent lowering of blood pressure and concluded that the developed NPs resulted in gastrointestinal stability and good anti-hypertensive efficacy. Likewise, amiodarone, an antiarrhythmic drug along with β-cyclodextrin was encapsulated in chitosan NPs, resulting in sustained release of the drug [98]. 

Wu et al. fabricated folate-functionalized NPs with an aim to overcome the lack of efficacy of the neat drug in rheumatoid arthritis. In vitro study showed increased cellular uptake of methotrexate encapsulated FA-GC-SA NPs via folate receptor mediated endocytosis and in vivo study revealed reduced proinflammatory cytokines. The results concluded that the developed NPs exhibited improved therapeutic efficacy. Subsequently, another study showed that eugenol, an antioxidant was encapsulated in chitosan NPs and these NPs demonstrated decrease in expression of TGF-β and MCP-1 genes assuring positive results in rheumatoid arthritis [99].

Petkar et al. successfully delivered rifampicin-loaded novel octanoyl chitosan NPs via pulmonary route for tuberculosis. The study indicated controlled drug release with excellent aerosolization properties. On similar lines, bedaquiline encapsulated in chitosan NPs were fabricated and evaluated against oral and dry powder inhalation of bedaquiline drugs. The nanoformulation was characterized by a better safety profile, higher therapeutic efficacy and improved target specificity [100].

In another study by Hill et al., tobramycin loaded alginate/chitosan NPs were formulated in an attempt to reduce the non-compliance factor of cystic fibrosis treatment. These NPs exhibited prolonged drug release and showed increased action on the thick mucus formed in the pulmonary environment due to the mucoadhesive nature of chitosan. Further, functionalizing the NPs with secretory leukocyte protease inhibitor (SLPI) showed characteristic inhibition of inflammatory response in lung infection.

## 9. Recent Developments in the Utility of Chitosan NPS

Conventional treatment of cancer includes chemotherapy, surgery and radiotherapy leading to various side effects with the anticancer drugs having poor aqueous solubility, failing to achieve tumor specific response and accumulate selectively at the site of action. Tumor-specific delivery is achieved by delivering the drugs in nanocarrier systems that help to improve the stability, biocompatibility and therapeutic efficacy of the drugs. Chitosan NPs are widely used nanocarriers as they are biocompatible, nonimmunogenic, nontoxic, target specific and can overcome the problems of low therapeutic index hydrophilic or hydrophobic drugs. Chitosan NPs facilitate controlled drug delivery (reduced dosage regimen) enhancing patient compliance to long term therapeutic regimen. The NPs localize and accumulate in tumors due to enhanced permeation and retention effect. Also, the tumors develop leaky capillary walls that eases the access of drug loaded NPs at the site, an important advantage over use of free drugs. This clearly indicates low toxicity of the drug loaded NPs in unaffected tissue and high therapeutic efficacy in tumors [101]. Additionally, NPs exhibit stability in blood circulation avoiding aggregation, premature drug leakage, easy removal by renal filtration and opsonization by reticuloendothelial system.

The drug molecules are linked to chitosan NPs along with a functional spacer that enables the nanocarrier system to show effective pharmacological activity at desired sites thereby reducing side effects [102]. The spacers are used to protect healthy organs from radiation exposure during nanoparticle therapy i.e., it preserves enough distance between the tumor and healthy tissue and is used for malignant tumors. 

The target specific delivery of the anticancer agent using chitosan-based NPs can be achieved by conjugation of tumor specific ligands on the chitosan nanoparticle. The ligands conjugated to NPs can easily interact with the cell surface receptors enabling receptor mediated endocytosis of the NPs. This technique works well as many surface receptors are overexpressed in cancer cells enabling receptor targeted delivery of chitosan NPs. The receptors commonly exploited for targeted delivery in cancer cells include epidermal growth factor receptor, folate receptor, CD44 receptor, integrins and low-density lipoprotein receptor. The expression of these receptors varies in different cancer types, so it is important to know the cancer cell type and receptor expression for achieving targeted drug delivery. Some recent research is reported here- Mohammadi et al. evaluated human serum albumin loaded chitosan NPs conjugated with MUC1 through an acrylate spacer for cancers overexpressing MUC1. The resultant chitosan NPs were found to be more effective and cytotoxic to tumor cells [103].

Chitosan fucoidan NPs loaded with pro-oxidant drug (piperlongumine) were evaluated for cancer therapy. The pro-oxidant agent had the ability to cause cancer specific apoptosis via increase in oxidative stress in cancer cells. The hydrophobic nature of piperlongumine limits this activity and incorporation in chitosan NPs results in increased water solubility and bioavailability. This study on PC-3 cells (prostate cancer cells) indicated significant cytotoxic effects for the NPs [104].

Chitosan covalently linked to L-cysteine and folic acid molecules entrapping methotrexate was evaluated for its clinical efficacy. The result of this study on HeLa cell line showed tumor specific drug delivery and sustained drug delivery due to overexpression of folate receptors and increased concentration of reductive agents (redox response) [105].

Recently, combinatorial NPs representing use of chemotherapy are being explored, for example- doxorubicin-cisplatin co-loaded NPs (hyaluronic and chitosan-based NPs) for breast cancer. It involves, cisplatin and doxorubicin conjugated to aldehyde hyaluronic acid by Schiff base reaction forming the inner core which is then subsequently complexed with hydroxyethyl chitosan and human epidermal growth factor receptor 2 antibody conjugated aldehyde hyaluronic acid. This resulted in both target specificity and enhanced cellular uptake leading to a useful synergistic cytotoxicity effect in human breast cancer MCF7 cells [106].

Chitosan is reported as a carrier for gene delivery in cancer therapy. Hyaluronic acid chitosan NPs encapsulated with cyanine3 labelled siRNA were conjugated with CD44 receptors for non-small cell lung cancer. The result showed that these NPs effectively delivered the siRNA to the target cells via CD44 receptor and inhibited cell proliferation by down regulating B-cell lymphoma 2 gene [107].

5-Fluorouracil encapsulated in hyaluronic acid chitosan NPs increased drug accumulation by transporting NPs to CD44- overexpressed tumor cells, resulting in apoptosis due to production of reactive oxygen species (ROS) [108]. Chitosan NPs are reported to be effectively used in both vaccine delivery and anticancer therapy promoting its utility in cancer immunotherapeutics.

### 9.1. Chitosan NPs in Cancer Immunotherapy

Immune system activation by using suitable adjuvants for improved therapeutic benefits is used in the treatment of several diseases especially cancer. These adjuvants stimulate the immune system and increase the response to a vaccine, without themselves being antigenic. The commonly used adjuvants in immunotherapy include aluminum salts like hydroxide and phosphate, lipopolysaccharide derivative, antimicrobial peptide, Toll like receptor agonist monophosphoryl lipid A and virosomes. Their use is associated with side effects, necessitating search of potential and safe adjuvants. Chitosan is used as an adjuvant due to its safety, biocompatibility and ability to act as an antigen carrier. Bioadhesive properties of chitosan promotes cellular uptake enabling mucosal immune response. It has the ability to enhance both humoral and cell mediated immune responses on antigen vaccination with clinical studies demonstrating improved properties of glycated chitosan versus chitosan for immunological stimulation [109]. It induces immune activity by stimulating phagocytic cells. It promotes IL-1 β secretion and induces mitochondrial DNA mediated cGAS-STING (cyclic GMP-AMP synthase stimulator of interferon genes) which results in secretion of interferon type 1. This stimulates maturation of dendritic cells resulting in antigen presentation and further stimulating type 1 T-helper immune response [110]. Characteristics of chitosan like molecular weight, degree of deacetylation, viscosity and endotoxin levels are important parameters in assessing the feasibility of chitosan-based adjuvant therapy.

Chitosan NPs are also used as adjuvant for DNA vaccines i.e., genetic immunization that includes co-formulation of genes with an immunostimulatory adjuvant, enhancing therapeutic efficiency of vaccine. Tahamtan et al. formulated chitosan NPs as an adjuvant for HPV-16 E7 DNA vaccine that induced antitumor immunity by genetic immunization. This study compared the co formulation (chitosan NPs + IL-12 with HPV-16 E7 DNA vaccine) and single vaccination with either chitosan NPs or IL-12. The results indicated that the co formulation showed an increase in IFN and IL-4 and decrease in IL-10 production and a significant increase in E7 specific lymphocyte proliferation index demonstrating higher antitumor effects for the co-formulation. The chitosan NPs proved to be an effective genetic adjuvant for eliciting enhanced antitumor immune response [111].

Maiyo et al. prepared a therapeutic mRNA nanocarrier system with a synergistic effect for cancer immunotherapy. This system comprised of chitosan functionalized-selenium NPs for folate targeted m-RNA delivery in cancer immunotherapy. The results of this study indicated that these NPs protected m-RNA from RNase degradation and these NPS were able to effectively deliver the m-RNA cargo in vitro, the incorporation of folate targeting moiety further increasing its uptake in folate receptor-positive cells. Their study proved that the use of chitosan-coated selenium NPs in mRNA delivery holds strong potential for application in cancer vaccination and immunotherapeutics [112].

### 9.2. Chitosan NPs in Theranostics

**Figure 5:**
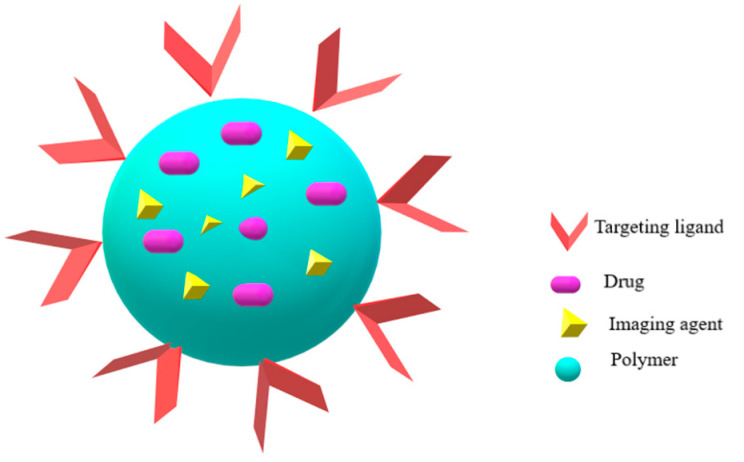
Chitosan NPs as theranostics.

Polysaccharide-based NPs are reported to be emerging platforms for simultaneous drug delivery and imaging as theranostic NPs for simultaneous or sequential administration. Chitosan NPs are amenable to development as theranostic agents as they have good loading capability, making ligand-based functionalization feasible. Figure 5 represents the theranostic features of chitosan NPs. It offers a wide range of derivatives that can be harnessed to functionalize imaging and therapeutic agents by chemical conjugation, cross-linking and/or charge-charge interactions. This allows the NPs to locate and reach the exact disease site and help identify the stage of the disease, its spread and response to treatment making customized patient specific therapy possible. Theranostics is used for monitoring drug delivery, release and for imaging by use of magnetic resonance imaging [113]. In theranostics, therapeutic and imaging agents are covalently conjugated with the functional groups of chitosan or physically encapsulated into chitosan NPs and can be used in image-guided photodynamic therapy. Chitosan NPs offer attractive features of nontoxic nature, biodegradability, biocompatibility, bioadhesiveness, physiological stability and decreased uptake by mononuclear phagocyte system prolongs the NPs residence time and increases accumulation at diseased sites enabling chitosan and other polymeric NPs to be used as a theranostic technologies. This reflects the wide scope of NPs to be used as co-delivery strategies in combination with diagnostics, prognostics and image guided therapy for improved therapeutic effects [114,115].

A recent review reported the application of chitosan NPs in therapeutics and theranostics of hepatocellular carcinoma (HCC) to overcome the limitations of conventional HCC therapies [116]. NPs were prepared with specific ligands enabling liver targeting and the results showed improved inhibition of tumor growth combining the benefits of diagnosis at early disease stage along with improved therapeutics. Functionalized chitosan NPs can be conjugated with therapeutic agents (like small molecules, proteins, peptides) and diagnostic agents (like imaging agents, photosensitizers, sensors) enables high performance NPs. For example: chitosan-hyaluronic acid-based NPs [117].

5-Aminolevulinic(5-ALA) is known to induce fluorescence and is used for photodynamic detection of the tumor cells. It is administered in engineered chitosan-based NPs in combination with alginate and folic acid for revealing colorectal cancer (CC) cells and is based on a light mediated mechanism. The NPs are endocytosed by the CC cells via folate receptor. Subsequently, the deprotonated alginate led to low desirability change between 5-ALA and chitosan resulting in charged 5-ALA giving rise to protoporphyrin IX (PpIX) for photodynamic detection within the cells [118]. There are many reports of chitosan NPs developed as theranostic systems that encapsulate the drug and carry the diagnostic or imaging agent on its surface demonstrating the growing landscape of chitosan NPs as theranostic systems especially the functionalized chitosan like glycol or PEG-chitosan with superior suitability for in vivo translation [117].

## 10. Conclusions

Nanocarriers have evolved to be attractive platforms to deliver drugs in a variety of diverse diseases with special emphasis on cancer due to their enhanced permeation and retention attributes. These nanocarriers offer the advantages of improved drug solubility, higher loading capacity and efficiency of drug payloads along with controlled drug release. Inorganic and organic nanocarriers have been extensively reported for developing systems with unique advantages, however the main limitation lies in controlling the particle size to sub 100 nm range with chemical functionalization strategies used to confer better drug loading and release kinetics adding to the increase in particle size. Additionally, the inorganic metallic NPs are associated with low biocompatibility and biodegradability despite providing high loading capacity. Organic polymeric NPs, especially chitosan-based systems confer desirable attributes of biodegradability, biocompatibility, permeation enhancement, mucoadhesiveness, antimicrobial, antifungal activity along with extremely useful efflux inhibitor property. Chitosan is reported to be used for decorating NPs via crosslinking and confers positive zeta potential offering better control of the physicochemical properties, changes in protein corona formation and enhanced cellular uptake resulting in improved drug efficacy. Its use as an adjuvant has opened up new vistas in immunotherapy, especially in the field of cancer. It’s potential as a theranostic is widely studied and reported by many research groups across the globe. Due to its versatility for application as a theranostic and drug delivery vehicle in cancer therapy, it is immensely studied in multi-stage drug delivery systems. These systems combine the dexterity and ability to diagnose, image and treat at the same time. Though there is an urgent need to examine its side effects and toxicity, in the near future, there is a huge untapped and emerging potential of chitosan NPs as multi-stage delivery systems in biomedical and pharmaceutical fields. The traditional tumor targeted DDS was based on enhanced permeability and retention (EPR) effect and receptor mediated endocytosis. However, now the emergence of multi-stage systems would provide a triple punch effect with enhanced tumor specific targeting. These systems also known as stimuli-responsive delivery systems respond to the extracorporeal physical stimuli and the endogenous tumor microenvironment resulting in various changes in size, zeta potential and carrier disassembly of NPs resulting in deeper penetration in tumor cells and enhanced targeting. The scope of future research lies in development of novel functionalized chitosan NPs based on computational studies that can predict the targeting potential and interaction with various proteins and signaling cascades overexpressed in cancer cells that would eventually culminate into lower drug dose, simpler dosing regimen and reduced side effects with better patient compliance and improved clinical outcomes.

## Figures and Tables

**Table 1:** Chitosan NPs- drug payloads, preparation, entrapment efficiency, advantages.

Diseases	Drug Payloads in Chitosan NPs	Preparation Methods	Entrapment (EE) Loading Efficiency (LE)	Release Profile	Advantages of Drug Loaded Chitosan NPs	Limitations of Neat Drug	References
Breast cancer	Methotrexate-loaded chitosan-modified mesoporous silica NPs	Cross-linking of mesoporous silica NPs (modified with 3-aminopropyl triethoxysilane by glutaraldehyde	EE = 12.2%	58.0% at pH 6.5	1—Amine modified NPs improved drug loading 2—Sustained drug release	1—Unmodified MSN poor drug loading 2—Poor burst release	[25]
Breast cancer	Melatonin-loaded pH-sensitive chitosan/ Hydroxypropyl- methylcellulose composite NPs	Cross Linking	NA	61% at pH 5.5	NPs exhibited higher toxicity for MDA-MB-231 cancer cells	Low efficacy	[26]
Oral cancer	Oxaliplatin-loaded chitosan NPs	Iontophoresis	-	-	1—Increased bioavailability 2—Increased drug penetration	Poor drug target specificity and penetration	[27]
Bladder cancer	Chitosan-hyaluronic acid dialdehyde NPs for CD44-targeted siRNA delivery	Ionotropic gelation	LE = >95%		Low cytotoxicity	Lack in target specific drug delivery	[28]
Cancer	Amygdalin-loaded alginate-chitosan NPs	Polyelectrolyte complexation	EE = 90%	86.03% at pH 7.4 after 10 h	1—Improved cytotoxic profile 2—Sustained Drug release	Presence of cyanide group-toxicity	[29]
Cancer	5-Fluorouracil-loaded chitosan/dextran sulfate/chitosan NPs for dual drug delivery	Double emulsion crosslinking method	EE: CS/DEX/CS NPs (66.3%) > CS/DEX NPs (62.4%) > CS NPs (57.3%)	5-Fu accumulation was 99.41% at pH 5.67 after 150 h And 32% for PTX after 150 h	1—Increased entrapment efficiency 2—controlled release 3—Enhanced inhibition in cancer cells 4—synergistic effects by dual drug delivery	-	[30]
Cancer	Chitosan-mediated solid lipid NPS for delivery of zedoary turmeric oil (ZTO)	Emulsion-solvent evaporation, thin film-ultrasonic dispersion	EE = 43 %	80% after 48 h	1—Efficient drug delivery to liver 2—Improved Bioavailability 3—Enhanced stability of ZTO	volatility, insolubility, low bioavailability	[31]
Cancer	Doxorubicin-aptamers-chitosan-gold NPs complex		EE = 85%	57% after 72 h at pH 5.5	1—Enhanced tumor inhibitory effect 2—Less distribution to other organs	Doxorubicin alone less tumor inhibitory effect Non-specific distribution across other organs	[32]
Infantile hemangioma	Propranolol hydrochloride- loaded lecithin/ chitosan NPs	double emulsion technique	EE = 53.62%	56.11% after 24 h	1—Minimum side effects 2—Optimum topical therapeutic concentration 3—Sustained drug release	Systemic adverse side effects	[33]
Antimicrobial and anticancer effects	Naringenin (NRG), quercetin (QE) and curcumin (CUR)- loaded chitosan-cellulose hydrogel conjugated with L-histidine and zinc oxide hybrid NPs	Crosslinking	LE: NRG-90.55% QE-92.84% CUR-89.89%	70.32% (QE), 77.54% (NRG) and 65.19% (CUR) at pH 5.0	Prominent antimicrobial activity against Staphylococcus aureus and Trichophyton rubrum strains due to synergistic effect Anticancer activity: A431 cells exhibited excellent cytotoxicity	polyphenol drugs show poor antimicrobial & anticancer activity	[34]
S. Pneumoniae infections	Cpl-1-loaded chitosan NPs	Ionotropic gelation method	EE = 55%	72.6% after 24 h	1—Increased bioavailability and half-life in-vivo 2—Chitosan NPs biocompatible candidate for Cpl-1 delivery	Low bioavailability	[35]
Antimicrobial activity against multidrug- resistant Staphylococcus aureus	*N*’-((5-nitrofuran-2-yl) methylene)-2-benzo-hydrazide was incorporated in polysorbate 20 micelles and further loaded in chitosan NPs (CH-5-NFB-NP),	Ionic gelation	EE = 44%	-	1—Enhanced inhibition of bacterial growth 2—promising results against multi drug resistant strains 3—Easy incorporation of hydrophobic drug	1—Drug resistance 2—Hydrophobic drug	[36]
Antibiotic	Gentamicin-loaded PLGA Chitosan NPs	Double emulsion method	EE = 92.5%	74–83% after 8 h	Oral Gentamicin nano formulation showed better therapeutic effects.	No oral route due to enzymatic degradation and poor bioavailability	[37]
Acne vulgaris	Clindamycin-loaded chitosan NPs	Ionotropic gelation method	EE = 42%	65% after 24 h	1—Targeted delivery to pilosebaceous units 2—Enhanced drug distribution profile	Poor drug penetration	[38]
Postoperative endophthalmitis	Glycol chitosan/oxidized hyaluronic acid hydrogel film with dual release of dexamethasone (Dex) and levofloxacin (Lev)	-	-	Lev—100% release in 10 min Dex—18% in first burst release	1—stepwise release of Lev and Dex, Lev—rapid release Dex—prolonged release 2—Potent anti-inflammatory, downregulation of inflammatory cytokines	-	[39]
Glaucoma, Ocular delivery	Tetrandrine lipid NPs (TET-LNPs)-loaded carboxy-methylchitosan (CMC), hydroxypropylchitosan (HPC) and trimethyl- chitosan (TMC)	Emulsion droplet method	TMC-TET-LNPs- EE% = 90.65%	76.1% of TET after 12 h	increased ocular retention time and bioavailability	Restricted ocular bioavailability	[40]
Glaucoma ocular delivery	Dorzolamide-loaded chitosan decorated polycaprolactone NPs	Single step emulsification method	EE = 72.48%	62.84% to 82.34%	1—Improved efficacy and safety 2—Sustained release	-	[41]
Trans- cutaneous immunization, transdermal delivery	Poly (DL-lactide-co-glycolide) (PLGA) NPs Antigen used: hen egg-white lysozyme (HEL)	Anti-solvent diffusion method	EE = 64.3%	NA	Iontophoresis and NP efficiently delivered HEL Antibody titers in greater concentration	Subcutaneous injection of HEL solution showed low accumulation in hair follicles	[42]
Immuno- therapeutic agents	Low molecular weight chitosan NPs for CpG oligodeoxy- nucleotide delivery	Ionic gelation method	EE = 88.09% to 97.34%	NA	1—Improved binding ability and intracellular uptake 2—Efficient immunostimulatory effect	Effective intracellular delivery is challenging	[43]
Migraine, intranasal & intravenous	Sumatriptan succinate-loaded chitosan NPs	Ionic gelation technique	EE = 59.60%	58% after 24 h	Target specific drug delivery	limited systemic availability of sumatriptan via oral administration	[44]
Parkinson, nasal delivery	Ropinirole Hydrochloride-loaded chitosan-coated PLGA NPs	Nano-precipitation method	LE = 5.7%	89%	Intranasal delivery surpasses hepatic metabolism and passes BBB to deliver Ropinirole.	1—High first pass metabolism 2—lower bioavailability	[45]
Gene therapy, intranasal delivery	siRNA-loaded chitosan NPs	Polyelectrolyte complexation method	-	-	1—Enhanced stability 2—Noninvasive gene therapy	Low stability	[46]
Scorpion Envenoming therapy, vaccine delivery	Chitosan NP encapsulating Aah II toxin	Ionotropic gelation	EE = 96.66%	Burst effect release of 55.37% in 8 h & 80.16% after 120 h	1—Enhanced immunization 2—Elicitation of systemic innate and humoral immune responses	-	[47]
Polycystic kidney disease, Oral delivery	Metformin-loaded chitosan NPs	Ionotropic gelation method	LE = 32.2%	>50% at pH 6.5	1—Reduced off target side effects 2—Improved Therapeutic efficacy 3—Increased oral bioavailability	Poor bioavailability	[48]
Peptide Macromolecule delivery via oral route	Octreotide-loaded pre-activated thiolated chitosan NPs	Ionic gelation	EE% = 85% to 91%	TCSNPs shows 50% after 6 h	1—Pre-activated thiomers prevent the oxidation of -SH group 2—Controlled drug release	Oxidation of -SH group, decreased efficacy	[49]
Diabetes, oral delivery	Insulin-loaded thiolated chitosan NPs	Microemulsion method	EE = 79.63% at pH 5.3	92% (after 24 h) at pH 5.3	1—Oral delivery-better patient compliance 2—Increased bioavailability due to interaction with the mucosal membrane of intestine 3—Prolonged drug delivery	Poor patient compliance due to subcutaneous route of injection	[50]
Diabetic wound healing	*Pterocarpus marsupium* heartwood extract/chitosan NPs (PMCNPs)-loaded hydrogel (PM-CNPsH)	Ionic gelation	-	44% in 17 h of PM-CNPsH and 38% in 15 h	1—Quicker wound healing in diabetes 2—Sustained Release	-	[51]
Wound healing and improvement in blood circulation	Chitosan-bromelain NPs	Ionic crosslinking	EE = 85.1%	-	1—Reduced the degradation caused by protease immobilization 2—Freeze-dried-improved stability	1—Unstable, suffers autolysis 2—Unstable when stored as aqueous suspension	[52]
Rheumatoid arthritis	Meloxicam-loaded chitosan-magnetite nanoconjugates	Co-precipitation	EE = 82%	98% after 6 h	Enhanced regional bioavailability, reducing dose frequency and dose related toxicity	Low bioavailability	[53]
Antioxidant	Resveratrol-loaded zein chitosan NPs	-	EE = 91%	-	1—Improved storage stability of Resveratrol 2—Sustained release	Poor stability on storage	[54]
Antioxidant peptides administration	Goby fish protein hydrolysate encapsulated in blue crab chitosan	Ionic gelation	EE = 58%	60.84%	1—Enhanced thermal stability 2—Diffusion-controlled mechanism 3—Improved antioxidant activity	Poor thermal stability	[55]
Hyper-lipidemia	Sodium alginate/polyvinyl alcohol hydrogel containing rosuvastatin-loaded chitosan NPs	Ionic gelation method	-	67% after 8 h and then slow release of only 20% between 8–24 h	1—Optimal mechanical properties 2—Controlled drug release	-	[56]
Prolonged blood circulation time of vitamin K1	Chitosan NPs loading VK1 adsorbed onto red blood cells	Ionotropic gelation	EE = 78.17%	80% after 10 days	1—Sustained release and prolonged circulation time of vitamin K1 2—Circulation time of RBC-hitchhiking chitosan NPs greater than regular NPs	Rapid clearance from circulation by mononuclear phagocyte system	[57]

**Table 2:** Drug release mechanism of chitosan NPs.

Swelling	Diffusion	Erosion
Drug release is controlled by degree of swelling.	Drug release is controlled by diffusion down the concentration gradient.	Drug release is controlled by physical or chemical degradation(erosion) of a polymer drug delivery system.
Water imbibition allows the drug to diffuse out.	This is based on Fickian model.	This can be due to surface or bulk erosion.
This can be linear for a short duration at interface.	This can be matrix-based or reservoir-based.	This depends on the surrounding medium or based on the presence of enzymes.

**Table 3:** Chitosan NPs in neurological diseases.

Diseases	Drug in Chitosan NPs	Zeta Potential & Loading Efficiency	References
Epilepsy	Carboxymethyl chitosan NPs as a carrier to deliver carbamazepine (CBZ-NPs)	Zeta potential of −32.1 with EE of 81.92%	[75]
Schizophrenia	Sulpiride-loaded chitosan NPs	EE and LE of 92.8% and 28% respectively	[76]
Parkinson’s disease	Rotigotine-loaded Chitosan NPs	Zeta potential (25.53 mV), and EE (96.08%)	[77]
Alzheimer	Galantamine-loaded thiolated chitosan NPs	-	[78]
Antipsychotic	Olanzapine-loaded chitosan NPs	The EE and LE was found to be 72.42% and 26.04	[79]

**Table 4:** Chitosan NPs in infectious and inflammatory diseases.

Diseases	Drug in Chitosan NPs	Zeta Potential & Loading Efficiency	References
Chronic Osteomyelitis	Vancomycin-loaded *N*-trimethylchitosan NPs	Zeta potential of 14.6 mV & LE of 73.65%	[80]
Atrophic Vaginitis	Chitosan ascorbate NPs loaded with amoxicillin trihydrate	-	[81]
Inflammatory Bowel Disease	Rifaximin-chitosan NPs	Zeta potential of 37.79, EE of 73%	[82]
Periodontitis	Tetracycline and lovastatin by poly (d,l-lactide-co-glycolide acid)-chitosan NPs	-	[83]
Post-operative eye inflammation (after cataract surgery) and allergic conjunctivitis	Chitosan-loaded ketorolac tromethamine NPs	Zeta potential of −21.8 & EE of 61.65%	[84]

**Table 5:** Chitosan NPS in diabetes and diabetes induced conditions.

Diseases	Drug in Chitosan NPS	Zeta Potential & Loading Efficiency	References
Diabetes induced retinopathy	Scutellarin-loaded amphiphilic chitosan derivatives (Chit-DC-VB12)	Zeta potential of 16.5 mV & LE of 13%	[85]
Diabetic wound healing	Curcumin-loaded chitosan NPs impregnated into collagen-alginate scaffolds	Zeta potential of 30.3 mV	[86]
Diabetes	Insulin-loaded thiolated N-triethyl chitosan NPs	Zeta potential of 24.6 mV & EE of 97.8%	[87]

**Table 6:** Chitosan NPs in vaccine delivery.

Diseases	Drugs in Chitosan NPs	References
Leishmaniasis	Chitosan NPs loaded with whole and soluble Leishmania antigens	[90]
Influenza	Killed SwIAV H1N2 (δ-lineage) antigens (KAg) encapsulated in chitosan NPs	[91]
Hepatitis B	Poly-ϵ-caprolactone/chitosan NPs provide strong adjuvant effect for hepatitis B antigen	[92]

**Table 7:** Chitosan NPs in miscellaneous diseases.

Diseases	Drug in Chitosan NPs	Zeta Potential & Loading Efficiency	References
Hypertension	ACE-inhibitory biopeptides encapsulated in chitosan NPs	Zeta potential of 48.78 mV & EE of 75.36%	[94]
Rheumatoid arthritis	Folic acid (FA) conjugated glycolchitosan (GC) NPs (FA-GC-SA) encapsulating MTX (methotrexate)	-	[95]
Tuberculosis	Rifampicin-loaded octanoyl chitosan NPs	EE of 64.86%	[96]
Cystic Fibrosis	Tobramycin-loaded alginate/chitosan NPs	Zeta potential of 21.6 mV & EE of 44.5	[97]
